# Could antidepressants increase mood and immunity at the same time?

**DOI:** 10.3389/fpsyt.2025.1340179

**Published:** 2025-03-12

**Authors:** Francis Lavergne, Therese M. Jay

**Affiliations:** Université Paris Cité, Institute of Psychiatry and Neuroscience of Paris (IPNP), INSERM U1266, Pathophysiology of Psychiatric Disorders, Paris, France

**Keywords:** antidepressant, acid sphingomyelinase enzyme, immunity, infection, stress amitriptyline, doxepine, fluoxetine

## Abstract

A review of scientific literature suggests that the use of antidepressants can be broadly extended to address various forms of stress and inflammation as an adjunctive therapy that enhances host resistance. While the effects of antidepressants on mood are well-documented in terms of their emotional, cognitive, and behavioral impacts, these aspects do not fully explain their cellular mechanisms of action. At the cellular level, antidepressants exert trophic effects that promote neurogenesis and synaptic connectivity. Studies demonstrate that antidepressants improve cell survival, enhance stem cell proliferation, and reduce danger perception (mood effects) in depressed patients and animal models of depression. These trophic properties highlight a deeper biological mechanism beyond their mood-related benefits. The acid sphingomyelinase (ASM) theory of mood offers a more compelling explanation of the cellular effects of antidepressants compared to the monoamine hypothesis. Antidepressants functionally inhibit the ASM enzyme, thereby reducing the production of ceramide, which directs cells toward increased survival, cytoprotection, and reproduction, as well as improved mood. This review also highlights research demonstrating that antidepressants enhance host resistance to infections, immunological challenges, stress, and depression. These findings support the potential use of antidepressants to bolster host resilience in scenarios involving infections, vaccinations, cellular aggression, stress, depression, and even aging.

## 1 Increase mood with antidepressants

In this article, we examine all commercially available chemical antidepressants. Treatments such as electroconvulsive therapy, repetitive transcranial magnetic stimulation (rTMS), sleep deprivation, light therapy, and physical exercise are excluded from the scope of this study.

**Table 1** provides a comprehensive list of the antidepressants included in this study along with their pharmacological mechanisms of action.

**Table 1:** List of antidepressants mentioned in the study along with their main pharmacological mechanisms of action.

INCREASE 5-HT	DO NOT INCREASE 5-HT
**MAOIs:** Deprenyl, phenelzine, moclobemide, hypericum perforatum	**Glutamate/AMPA:** Ketamine
**SNRIs/TCAs:** Venlafaxine, amitriptyline, desipramine, amoxapine, clomipramine, impramine, nortriptyline, doxepine, lofepramine, trimipramine	**NRIs, NDRIs:** Bupropion, reboxetine, maproptyline
**SSRIs:** Citalopram, escitalopram, fluvoxamine, fluoxetine, paroxetine, sertraline, norfluoxetine, zimeldine	**5HT2a antagonists:** Mianserine, mirtazapin
**Others:** Trazodone, lithium chloride and carbonate	**Others:**

Conventional antidepressants act by regulating monoamines in the central nervous system, increasing serotonergic and noradrenergic transmission. **Table 1** divide antidepressants into those that increase serotonin (5-HT) and those that do not increase serotonin (5-HT) based on their primary mode of action.

MAOIs, Monoamine oxidase Inhibitors; SNRIs, Serotonin–norepinephrine reuptake inhibitors; TCAs, Tricyclic antidepressants; SSRIs, Selective serotonin reuptake inhibitors; Glutamate/AMPA, Glutamate release increase and AMPA receptor activation; NRIs, Noradrenaline reuptake inhibitors; NDRIs, Noradrenaline dopamine reuptake inhibitors; 5HT2a antagonists, Blockade of 5HT2a receptors.

We understand that all antidepressants exhibit comparable efficacy in treating depression. Head-to-head trials of 21 antidepressants versus placebo in major depressive disorder (MDD) report odds ratios ranging from 2.13 (95% credible interval: 1.89–2.41) for amitriptyline to 1.37 (95% credible interval: 1.16–1.63) for reboxetine [with 21 antidepressants, (1)].

The efficacy of antidepressants is observed both clinically, through improvements in patients’ mood, and via standardized depression scales. At the cellular level, these medications promote pro-life processes, including cell reproduction, cytoprotection, and survival. These mechanisms serve as hallmarks of the antidepressant effect at the cellular level. All antidepressants, whether administered orally (per os) or intravenously (IV), enter the bloodstream and reach nearly all cells in the body. The resulting mood changes are reflected across three dimensions: (1) the emotional dimension with a reduction in sadness, fear, and disgust, along with a diminished perception of danger (2) a cognitive dimension with alleviation of impairments in memory, concentration, and learning (3) a behavioral dimension with decreased psychomotor retardation and anxiety, alongside improvements in communication and overall functionality.

At the cellular level, mood improvements are associated with a neurotrophic state, characterized by increased neurogenesis and enhanced synaptic connectivity. Antidepressants elevate mood and promote a trophic state in cells by boosting cell reproduction (e.g., neurogenesis), enhancing cell resistance (e.g., cytoprotection), and increasing survival against toxic and infectious challenges.

Antidepressant-like effects have been observed in animal experiments (e.g., rats and mice) as early as 20 minutes after intraperitoneal administration. In humans, behavioral changes can be detected within two hours of an antidepressant injection. For instance, healthy individuals administered citalopram exhibit greater prosocial behaviors, enhanced positive recall, and improved positive learning of social evaluations toward others (2).

Evidence from the Zurich meta-analysis indicates that the effect of antidepressants on mood begins to diverge significantly from placebo after only five days of treatment [e.g., tricyclic antidepressants versus placebo, (3)]. Similarly, a naturalistic study of 4,771 depressed patients demonstrated a one-point improvement on the Clinical Global Improvement Scale after just one week of antidepressant therapy. Montgomery-Åsberg Depression Rating Scale (MADRS) scores show a 2.5% daily reduction during the first two weeks of treatment, followed by a slower decrease of 0.5% per day from week two to week six [e.g., mirtazapine, (4)].

Overall, most antidepressant-related mood changes occur during the first two weeks of treatment [e.g., tricyclics, (3); with mirtazapine, (4)].

## 2 Increase host-resistance with antidepressants

### 2.1 Increase Survival with Antidepressants

#### 2.1.1 Infections

The above findings are supported by observations of improved survival in animals receiving antidepressant treatment during viral infections [e.g., fluoxetine, (5)], bacterial infections [e.g., amoxapine, (6)], or LPS-induced septic shock [e.g., desipramine and fluoxetine, (7)].

These observations have been further validated in humans with COVID-19. Studies have shown that infected patients receiving antidepressants, such as fluoxetine, paroxetine, escitalopram, venlafaxine, mirtazapine (8); fluvoxamine (9–13); trazodone (14); citalopram (15); and other selective serotonin reuptake inhibitors (SSRIs), serotonin-norepinephrine reuptake inhibitors (IRSNa), or mirtazapine (16), experienced slower clinical deterioration and reduced mortality compared to patients not receiving antidepressant therapy. Additionally, an observational study investigating fluoxetine, fluvoxamine, escitalopram, amitriptyline, and other FIASMA [functional inhibitors of acid sphingomyelinase, (17)] antidepressants supported these findings. Another study involving 22 different antidepressants demonstrated similar protective effects (18).

Preclinical studies further reinforce these results. Fluoxetine has been shown to replicate its antiviral and anti-inflammatory activities in rats infected with SARS-CoV-2, consistent with its effects observed in humans (19). The magnitude of antidepressant-associated survival benefits in animal studies is striking. For example, in a model of bubonic plague, mice were administered eight times the lethal dose (LD50) of Yersinia pestis and treated with or without an antidepressant [e.g., amoxapine, (6)]. More than 50% of the antidepressant-treated mice survived beyond day 21, whereas all untreated mice succumbed within four days.

(**Figure 1**).

**Figure 1:**
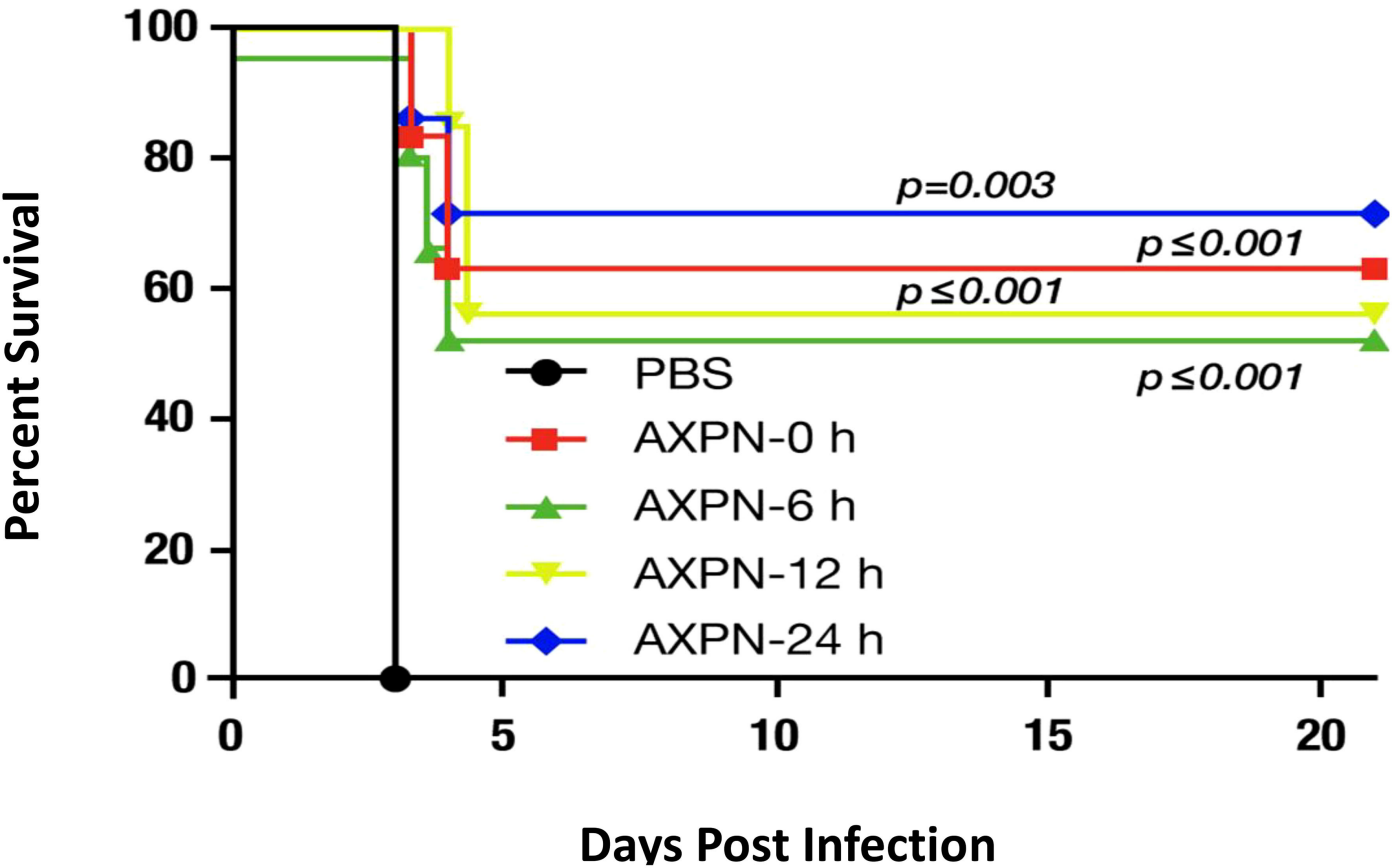
Effect of amoxapine (AXPN) treatment on the survival of mice infected intranasally with *Yersinia pestis* (WT CO92) at a dose of 8 LD50. Four AXPN treatments (3 mg/Kg; n=7) are administered intraperitoneally for 3 days at 24-hours intervals. These treatments start at the time of infection (AXPN-0), 6 hours after infection (AXPN-6), 12 hours after infection (AXPN-12), and 24 hours after infection (AXPN-24). The control group receives phosphate-buffered saline (PBS; n=5). The data were analyzed using Kaplan-Meier for significance to compare the AXPN group to the control group. Adapted from Andersson et al., 2017; Combating Multidrug-Resistant Pathogens with Host-Directed Nonantibiotic Therapeutics (6).

The authors verified that antidepressants have no direct bactericidal effect on bubonic plague; instead, they enhance host survival. In another study involving lipopolysaccharide (LPS) exposure in mice, two antidepressants [desipramine and fluoxetine, (7)] were compared to prednisolone. Similar survival rates were observed with prednisolone (30%) and the two antidepressants (30% and 50%), whereas the control group (no treatment) showed a 90% mortality rate. These findings demonstrate a significant survival benefit with antidepressant treatment during infection, corresponding to a 40–60% increase in survival.

This survival benefit can be generalized to various types of infections: viral infections: [e.g., amitriptyline, (20); antidepressants used by patients, (21)], bacterial infections: [e.g., amitriptyline, (22, 23); fluvoxamine, (24)], parasitic infections: [e.g., desipramine, (25)], fungal infections: [e.g., sertraline, (26, 27); fluoxetine, (28)].

At the cellular level, antidepressants reduce tissue damage caused by stress and infection. This protective effect has been observed across all forms of inflammation, including autoimmune disorders: [e.g., amitriptyline, (29); fluoxetine, (30); phenelzine, (31); St. John’s wort, (32); imipramine, (33); clomipramine, (34); paroxetine, (35); amitriptyline, (36); zimeldine, clomipramine, imipramine, maprotiline, (37)], toxic aggression: [e.g., mirtazapine, (38)], traumatic injuries: [e.g., amitriptyline, (39)], spinal cord injury: [e.g., fluoxetine, (40, 41)], wound healing: [e.g., fluoxetine, (42)], and burn injuries: [e.g., citalopram, (43)].

These findings highlight the broad protective effects of antidepressants in mitigating inflammation, reducing lesions, and improving survival during various pathological conditions.

The protective effects of antidepressants may involve the ASM/ceramide pathway. Sphingomyelinases and ceramide play crucial roles in various infection-related processes, including the internalization of pathogens, the induction of apoptosis in infected cells, the activation of intracellular signaling pathways, and the release of cytokines. These diverse functions highlight ceramide as a key player in the host response to numerous pathogens (44). Peripheral inflammation, characterized by increased ceramide levels in the blood, can impact regions of the brain in direct contact with the bloodstream, such as the sub-granular zone of the dentate gyrus in the hippocampus, the subventricular zone of the lateral ventricles, and the hypothalamus (45).

Beyond these effects, antidepressants actively promote cell replication and provide cellular protection both *in vitro* and *in vivo*.

### 2.2 Cells replication

The proliferative effect of antidepressants is both evident and underappreciated. It is evident because all classes of antidepressants enhance neurogenesis by stimulating neuronal stem cell proliferation [e.g., SSRIs, tricyclics, and MAOI, (46)]. However, it remains underappreciated because their effects extend beyond neuronal stem cells, also promoting proliferation in autologous stem cells [e.g., desipramine, (47)], adipose-derived stem cells [e.g., fluoxetine, (48)], and endometrial stem cells [e.g., citalopram, (49)].

Adding an antidepressant to mesenchymal stem cell cultures significantly increases cell proliferation. For instance, a 30-day incubation with sertraline and retinoic acid results in 7.41 population doublings compared to only 5.63 doublings without sertraline (50). Since immune cells originate from hematopoietic stem cells in the bone marrow, the enhanced proliferation induced by antidepressants could also boost immune system efficacy. Supporting this hypothesis, antidepressants have been shown to increase the efficacy of vaccinations, such as the zoster vaccine, with better immune responses observed in treated individuals (51). The proliferative effects of antidepressants influence neurons, the immune system, and potentially all cell types in the body.

### 2.3 Increase cells protection

The revolutionary aspect of antidepressants lies in their demonstrated ability to protect cell cultures from dying. Astonishingly, this protection is not limited to enhancing host resistance but extends to a direct cytoprotective effect at the cellular level. In non-neuronal cells, this effect occurs independently of neurotransmission. Antidepressants directly shield cells from toxic assaults, as observed with desipramine, fluoxetine, and moclobemide. In cell culture models exposed to high, toxic concentrations of corticosteroids, desipramine (0.625–10 µmol/L), fluoxetine (0.625–10 µmol/L), and moclobemide (2.5–40 µmol/L) exhibit significant cytoprotective effects (52). This phenomenon is also evident *in vivo*, as demonstrated in a rat model of diabetes where lithium reduces pancreatic β-cell damage caused by streptozotocin. Lithium provides cytoprotection and attenuates oxidative stress [e.g., lithium chloride, lithium carbonate, (53)]. The cytoprotective effects of antidepressants are further observed in wound healing. Studies show these effects in open wounds treated with topical fluoxetine (54), amitriptyline-based biodegradable PEG-PLGA self-assembling nanoparticles (55), fluoxetine gel in solid lipid nanoparticles (56), and other formulations of topical fluoxetine (57). In humans, these benefits extend to wound treatment using Hypericum perforatum (St. John’s Wort) (58).

However, this cytoprotective effect could be detrimental in scenarios requiring immunosuppression, such as during kidney allogenic grafts. Studies suggest that the use of antidepressants before transplantation might interfere with the desired immunosuppressive state (59). In some instances, antidepressants could inadvertently support both sides of an immune conflict: the host and the adversary, such as cancer cells or blood parasites.

For example, while antidepressants can enhance the host immune response, they may also promote cancer cell proliferation if cancer is already present. Research on this subject presents conflicting outcomes. On one hand, antidepressants appear to lower cancer risk within populations; on the other, they may encourage the growth of existing cancer cells (60). This dual effect underscores the complexity of their impact, warranting further investigation.

The ASM theory offers the most compelling explanation for this cytoprotective effect. ASM generates ceramide, a molecule that accumulates and induces mitochondrial and cell membrane damage, ultimately leading to apoptosis (61).

Antidepressants inhibit ASM activity, thereby preventing ceramide accumulation and protecting cells from apoptosis. Importantly, this inhibitory effect on ASM is a common feature across different classes of antidepressants, including MAOI, SSRIs, and tricyclic antidepressants [e.g., amitriptyline, fluoxetine, (62); tricyclic antidepressants, (63)].

## 3 Discussion

Our understanding is that antidepressants simultaneously enhance immune system efficacy and improve mood. This dual cellular effect of antidepressants holds clinical potential for boosting host resistance to infections and stress.

### 3.1 Clinical discussion: antidepressants enhance host resistance

Antidepressants are increasingly recognized for their role in enhancing host resistance against infections, including herpes virus infections [e.g., amitriptyline, nortriptyline, desipramine, (64)]. While the common understanding is that antidepressants primarily alleviate infection-related pain through their effects on neurotransmission, it is less appreciated that these drugs may also help patients combat the infection itself. This effect is potentially linked to a reduction in ceramide accumulation in the host [e.g., amitriptyline, (65)]. We propose that antidepressants not only enhance host resistance to stress but also bolster the host’s ability to manage all forms of infections, toxic aggressions, and even aging-related cellular damage [e.g., deprenyl, (66)]. This generalized effect positions antidepressants as valuable adjunctive therapies across a wide range of diseases. By augmenting host resistance, antidepressants may offer clinical benefits that extend beyond their traditional use in mood disorders.

### 3.2 Effects of Age

In both the immune system and mood regulation, we anticipate that antidepressants will yield greater benefits for aged and depressed patients than for young and healthy individuals. During the COVID-19 pandemic, mortality rates were strongly linked to the age and vulnerability of the patients. Notably, a clinical study on COVID-19 demonstrated that antidepressants can mitigate age-related vulnerability [with SSRI, (67)]. In this study, the antidepressant group was significantly older than the control group (p<0.001), yet the death rates were nearly identical (18% vs. 17%, respectively), indicating that antidepressant treatment effectively neutralized the impact of aging on COVID-19 outcomes. To further explore the age effect in host resistance, it is crucial to compare responses to antidepressants between older and younger subjects. We hypothesize that antidepressants will result in a greater survival benefit in aged populations due to their heightened vulnerability. Additionally, studies specifically targeting elderly populations could provide valuable insights into the role of antidepressants in enhancing host resistance, particularly in this high-risk demographic.

Some negative findings, such as the lack of effect of amitriptyline on wound healing [with topical amitriptyline, (68)], contrast with the demonstrated efficacy of fluoxetine (57) and St. John’s wort (58) in promoting wound healing when applied topically. This discrepancy might be explained by the use of young, healthy animals with optimal host resistance in the amitriptyline study. Antidepressants are likely to offer minimal benefits in young, healthy, and non-stressed subjects, as their host resistance and regenerative capacity are already at their peak (69).

Similarly, young and healthy individuals often show minimal mood-related effects from antidepressants.

In contrast, older and stressed subjects are expected to gain more significant benefits from the mood-enhancing and trophic effects of antidepressants (70).

These drugs could be prescribed as add-on therapies to bolster host resistance during vaccination, infections, and autoimmune conflicts. Furthermore, the influence of antidepressants on cell reproduction warrants exploration in the context of infertility treatment. However, the trophic effects of antidepressants also raise concerns about their potential to promote graft rejection and cancer progression. These possible risks highlight the need for careful evaluation of antidepressant use.

### 3.3 Pharmacological discussion: antidepressants increase host-resistance

The serendipitous discovery of the mood effects of antidepressants originated from their use as antibiotics. MAOIs were first used to treat tuberculosis, where antidepressant effects were observed in sanatoriums. Despite the cross-talk between neurons and immune cells, with both systems presenting the same type of receptors on the cell membranes, we have no understanding of the circuitry that goes from the antidepressant molecule to the effects on mood and immunity (71).

However, we have two pathways to explain the antidepressant host-resistance increase on mood and immunity.

#### 3.3.1 Classical mechanism of action of the antidepressant effect

The monoamine theory, widely endorsed by the pharmaceutical industry, posits that the augmentation of serotonin and/or noradrenaline neurotransmission is central to the antidepressant effect. Antidepressants target various neuronal and immune receptors (e.g., noradrenaline, serotonin, dopamine) to exert their effects through a complex network of interactions among neurons. This theory assumes the existence of a specific pathway that must be activated to enhance mood and neurogenesis. While numerous pathways have been explored, they tend to explain the side effects of antidepressants rather than their primary effects on mood and immunity. Our previous work aligned with the neurotransmission theory, showing that all antidepressants increase dopamine levels in the frontal cortex of rats [via chemical antidepressants, electroconvulsive therapy, repetitive transcranial magnetic stimulation, and sleep deprivation, (72)], regardless of their initial monoamine interactions with neuronal receptors. Furthermore, we demonstrated that a pure, selective D1 agonist alone could produce an antidepressant-like effect in the forced swim test [with A77639, (73)]. These findings shows that D1 receptor agonism induces a dose-dependent antidepressant efficacy in an animal model of depression. Interestingly, this D1 agonist was also shown to induce significant acidification of lysosomes, which functionally inhibits the ASM enzyme and reduces ceramide levels (74). This discovery introduces a second pathway that may explain how antidepressants enhance host resistance.

#### 3.3.2 The lipid-base pathway: ASM - ceramide - phosphatase

The ASM/Ceramide theory suggests that certain antidepressants functionally inhibit the enzyme ASM, leading to a reduction in ceramide production over a period of approximately one week. Research from the Gulbins group has highlighted the role of a lipid-based pathway in the pathology of depression, a pathway activated by psychosocial stress, oxidative stress, or inflammation. This overload of the ASM/ceramide pathway under stress conditions may explain mood disorders and their associated behaviors and inflammatory responses. The accumulation of ceramides is particularly detrimental, as it promotes inflammation and oxidative stress (75).

A recent study (76) offers compelling evidence for a novel conceptualization of the pathogenesis of MDD. It proposes that MDD begins with a peripheral increase in ceramides in response to stress and subsequently manifests in the brain. This reinforces the idea that MDD is not solely a brain disorder but affects the entire body. The study demonstrates three critical findings: (1) stress generates ceramides: Stress triggers the production of ceramides, which can initiate depressive symptoms, (2) ceramides induce depression: elevated ceramide levels are directly linked to the onset of depression, (3) antidepressants reverse depression and reduce ceramide levels.

##### 3.3.2.1 Stress generates ceramides

Exogenous stress triggers the release of ceramides into the bloodstream, as demonstrated in a mouse model under two conditions: (i) chronic unpredictable environmental stress and (ii) glucocorticoid administration through the water supply. In comparison to unstressed mice, ceramide levels were found to accumulate significantly in endothelial cells following the induction of stress. The measurement of ceramide levels was conducted using two independent methodologies—mass spectrometry and ceramide kinase assays. Both approaches consistently revealed a doubling of plasma ceramides in response to stress (76).

##### 3.3.2.2 Ceramide induces MDD

Clinically, patients suffering from moderate and severe major depression (Hamilton depression rating score ranging from 23 to 27 points for moderate and from 28 to 37 points for severe) show a marked increase (p<0.001) in ceramide concentrations in the plasma compared to healthy individuals. A second, independent set of older major depressive disorder patients (70 years old) and controls (68 years old) confirmed the finding of an increase in ceramide in the blood plasma of patients (76). Several other studies show that ceramide levels are increased in the blood plasma of patients with MDD (77–81) raising the question of whether ceramide is not only a marker of depression but might also play a role in the pathogenesis of depression.

**Experimental proof**. Ceramide Administration Induces Depression with its changes in neurogenesis and behaviors (76).

**Exogenous ceramide induces depression-like behavior**. Exogenous ceramide has been shown to induce depression-like behavior. In an experimental setup, plasma was isolated from the blood of untreated wild-type mice, loaded with a total of 5 nmol ceramide at a specific ratio, and subsequently injected into wild-type recipient mice. Following this treatment, a significant reduction in neurogenesis was observed, as assessed by counting BrdU-positive cells in the hippocampus and behavioral changes (recipient mice exhibited increased immobility in the forced swim test, alongside other indicators of depression-like behaviors). These findings provide compelling evidence that ceramide can induce both the behavioral and neurobiological hallmarks of depression in animal models, demonstrating its active role in the pathophysiology of depression (76).

**Ceramide in blood plasma injection**. The transfer of depression-like behaviors was demonstrated through the injection of blood plasma from stressed mice into non-stressed, healthy mice. The blood plasma from stressed mice contained elevated levels of ceramide. Upon injection into healthy mice, the recipient healthy mice exhibited depression-like behaviors, as evidenced by increased immobility in the forced swim test and other behavioral assessments. The observed changes were statistically significant (76).

**Stressed mice plasma incubated with anticeramide antibodies**. Anti-ceramide IgM antibodies (clone S58-9) or ceramidase (recombinant ceramidase) prior to re-injection into non-stressed mice prevented the development of depression-like symptoms, whereas control IgM or control IgG exerted no effect on the induced depression state (76).

##### 3.3.2.3 Commercialized ADs reduce ceramide and reverse depression (ASM/ceramide and membrane/synapse function)

Within cells, the ASM enzyme catalyzes the degradation of sphingomyelin into phosphorylcholine and ceramide. The accumulation of ceramide has detrimental effects, including increased oxidative stress, enhanced release of proinflammatory cytokines, and inhibition of hippocampal neurogenesis (82). These processes collectively contribute to the development of depression-like symptoms. In contrast, E. Gulbins et al. demonstrated that commercial antidepressants such as amitriptyline and fluoxetine reduce ceramide levels in the hippocampus of stressed mice, leading to increased neurogenesis and a reversal of depressive behaviors (83). E. Gulbins et al. further elucidated how antidepressants act on the ASM/ceramide pathway: conventional lysosomes can fuse with the plasma membrane in response to elevations in intracellular calcium (Ca²^+^), releasing their ASM content extracellularly (84). Antidepressants inhibit ASM activity, either at the cell surface or within lysosomes, reducing ceramide production. Ceramide molecules are known to alter membrane biophysics through their self-association within the cell membrane. This self-assembly leads to the formation of ceramide-enriched platforms, which cluster receptors such as the FAS (CD95) receptor. Activation of the FAS receptor initiates a signaling cascade involving caspases, ASM, ceramide, and JNK/p38-K, ultimately resulting in cell death (with tricyclic antidepressants, 64). Ongoing research explores how ceramides interact with cell membranes and monoamine receptors (85). The ASM/Ceramide theory proposes that antidepressants reduce ceramide accumulation, to produce mood and trophic effects, ultimately contributing to membrane stabilization and cell survival. This stabilization of cell membranes is crucial, as membranes facilitate communication with other cells. In the organism, communication occurs via hormones, the immune system, and the central and autonomic nervous systems. In neurons, synapses act as the central interface for cell-to-cell communication. Could the lipophilic ASM/ceramide pathway modulate synaptic function? Research by Kalinichenko and Kornhuber (85) supports this possibility, emphasizing the importance of lipid-protein interactions at synapses: “*The highly dynamic lipid composition of synaptic membranes suggests that these lipids and their interactions with proteins may contribute to the plasticity of the 5-HT synapse*.” This statement highlights the potential role of synaptic membrane lipids, including ceramides, in shaping the plasticity of serotonin (5-HT) signaling. Could the protective action of antidepressants, as observed in cell cultures and wound healing, operate universally on cell membranes and across all synapses?

**FIASMA and NO-FIASMA antidepressants**. By definition, FIASMA (functional inhibitors of acid sphingomyelinase) antidepressants exhibit residual activity of <50% on the ASM enzyme. Studies by Beckmann et al. [with tricyclic antidepressants, (63)] and Kornhuber et al. [with 19 antidepressants, (86)] highlight this characteristic. The residual ASM activities for FIASMA antidepressants is 11% for amitriptyline, 32% for imipramine, 21% for clomipramine, 15% for desipramine, 46% for doxepin, 19% for lofepramine, 13% for nortriptyline, 12% for protriptyline, 13% for trimipramine (**Table 2**).

**Table 2:** Table summarizing FIASMA and NO-FIASMA antidepressants, based on their residual activity (RA) on the acid sphingomyelinase (ASM) enzyme.

Antidepressants n=19	FIASMA <50% RA	NO-FIASMA >50% RA
amitriptyline	11%	
Clomipramine	21%	
trimipramine	35%	
bupropion		117%
doxepine	46%	
citalopram		79%
fluoxetine	13%	
maproptyline	13%	
mirtazapine		100%
norfluoxetine	22%	
nortriptyline	13%	
paroxetine	31%	
reboxetine		105%
sertraline	12%	
desipramine	15%	
fluvoxamine	37%	
imipramine	32%	
mianserine		111%
venlafaxine		95%

ASM activity after incubation for 30 minutes from Kornhuber et al. (86). Four out of six non-FIASMA antidepressants exhibit a residual ASM activity higher than that of control cells, indicating that they increase ASM enzyme activity. For instance, mianserine and mirtazapine enhance ASM activity compared to non-treated cells. Notably, both drugs possess a high antagonist affinity for 5HT2A and H1 receptors, with pKi > 9.

Not all antidepressants qualify as FIASMA (86). Certain antidepressants, such as mianserine, mirtazapine, bupropion, and reboxetine, present higher residual ASM activity than control. In fact, Kornhuber et al. (86) demonstrated that these antidepressants result in ASM activity greater than 100% compared to untreated cells, after 30 minutes of incubation. This means they enhance ASM activity, disqualifying them as FIASMA. Nearly all antidepressants exhibit one of two distinct effects on ASM activity: FIASMA antidepressants significantly inhibit ASM, with residual activity <50%. Non-FIASMA antidepressants increase ASM activity to levels >100% of the control. Interestingly, both FIASMA and non-FIASMA antidepressants can be equally effective in treating depression, though their mechanisms differ. This dual behavior suggests that antidepressants may act via diverse pathways, with ASM modulation being just one component of their broader pharmacological profiles (86) (**Table 3**).

**Table 3:** Four classes of antidepressant could be constructed.

5HT increase - ASM inhibition	No 5HT increase - ASM inhibition
Amitriptyline, doxepine, fluoxetine, maproptyline, norfluoxetine, clomipramine, nortriptyline, paroxetine, sertraline, fluvoxamine, imipramine, desipramine	

5HT increase - no ASM inhibition	No 5HT increase - no ASM inhibition
Citalopram, venlafaxine	Mirtazapine, mianserin, bupropion, reboxetine, trimipramine

Most antidepressants working with a serotonin increase in transmission are FIASMA. The NO-FIASMA property is associated with dopamine and noradrenaline modulation.

**Near total ASM inhibition**. Near-total inhibition of the ASM enzyme (<5% residual activity) has been shown to induce depression-like behaviors or to treat mania, depending on the context. Among 276 drugs studied (86), the two most powerful ASM inhibitors, with residual activity below 5%, are: (1) emetine with a residual ASM activity of 0.4% after 24 hours of incubation and (2) tamoxifene with a residual ASM activity of 4.1% after 30 minutes incubation. These drugs do not treat depression but instead induce depression-like behaviors and are effective in treating mania. Emetine is a potent inhibitor of protein synthesis, known to induce vomiting (87) and amnesia (88). At the cellular level, emetine modulates long term depression at the synaptic cleft (89) and induces 10 to 15% apoptosis in hepatocytes within 4 h (90). These cellular effects mimic the symptoms of depression, such as synaptic dysfunction and impaired communication. Without protein synthesis, synaptic function is effectively “knocked down,” disrupting normal neuronal signaling (91).

Tamoxifen is a partial agonist of the estrogen receptor commonly used in oncology. Remarkably, it has been identified as one of the most effective anti-manic drugs in two meta-analyses: one involving 25 anti-manic drugs (92) and another including 51 monotherapies, adjunctive treatments, or placebo (93). Near-complete inhibition of the ASM enzyme induces depression-like behaviors, while strong inhibition (11–50%) is characteristic of FIASMA antidepressants. Conversely, NO-FIASMA antidepressants appear to oppose ASM inhibition. In all cases, antidepressants interact with the lipidic pathway: either reducing ASM activity (FIASMA) or maximizing it (NO-FIASMA).

**All the synapses?** Clinically, depression can be studied through behavior, emotion, and cognition. Memory and learning are particularly sensitive indicators of mood. From a cognitive perspective, both memory and learning are diminished during episodes of depression and mania, while a euthymic state optimizes these cognitive functions. Cognitive synapses represent the most sophisticated form of information transfer facilitated by membrane contact. At the synaptic cleft, 5HT, dopamine, and potentially other neurotransmitters may modulate the signal. This perspective reconciles the monoamine and ASM theories: within the synapse, dopamine and 5HT collaborate with the lipidic pathway to fine-tune signal modulation. This process is not confined to the brain but likely occurs throughout the body, as 5HT and dopamine are actively transported by platelets (94).

At least two neurotransmitters play critical roles in regulating synaptic plasticity: (1) 5HT promotes the upregulation of functional AMPA receptor expression and triggers the rapid synthesis of AMPA receptors in motor neurites [as observed in *Aplysia*, (95)]. Within the synapse, protein synthesis is essential for local postsynaptic protein production, supporting learning-related synaptic plasticity mechanisms such as long-term potentiation and long-term depression. (2) Dopamine stimulates the synthesis of the GluR1 subunit of AMPA receptors in dendrites of hippocampal neurons in culture. Activation of dopamine D1/D5 receptors promotes local protein synthesis in hippocampal dendrites [with D1/D5 receptor agonists, (96)]. The interplay between 5HT, dopamine D1/D5 activation, protein synthesis, and membrane lipids in modulating synaptic function is an intriguing and promising area of research. These interactions may underlie key mechanisms of learning, memory, and synaptic plasticity.

All antidepressants exert their effects through at least one or more of the following mechanisms: (a) *5HT Transmission*. Many antidepressants enhance 5HT transmission, including classes such as IRSNa, SSRIs, tricyclic antidepressants, and MAOIs. (b) *Dopamine Release*. In studies using microdialysis in the frontal cortex of rats, all antidepressants have been shown to increase dopamine release (72). (c) *Functional Inhibition of ASM*. Some antidepressants functionally inhibit ASM, while others do not. This inhibition varies across different antidepressant classes and contributes to the lipid-based pathway involved in mood regulation and cellular protection. Our understanding suggests that 5HT, in conjunction with the lipid pathway, modulates synaptic activity, as proposed by Kornhuber and Kalinichenko for the 5HT synapse (85). This modulation occurs through interactions between membrane lipids and neurotransmitter receptors, which influence synaptic plasticity and communication. Furthermore, dopamine, glutamate, and brain-derived neurotrophic factor, combined with local protein synthesis, transform the 5HT signal into synaptic growth, facilitating long term potentiation and structural synaptic changes. In this framework, dopamine acts as a “shortcut” within the synapse, bypassing intermediary steps in the serotonergic pathway to directly promote plasticity and growth (97–99).

**Two questions related to antidepressant efficacy need to be tested**. *Is the FIASMA property essential for the direct protective effects of antidepressants on cell cultures*? From our review, it appears that FIASMA antidepressants consistently protect cell cultures from toxic aggression, whereas there are no reported findings indicating similar protective effects for NO-FIASMA antidepressants such as mianserin and mirtazapine. While this observation suggests a potential link between FIASMA properties and cellular protection, definitive evidence is lacking. All classes of antidepressants (FIASMA and NO-FIASMA) and mood regulators should be tested for their ability to protect cultured cells. The experimental framework could replicate findings from LI Yun-Feng et al. (52) using PC12 cells exposed to high concentrations of corticosterone (0.2 mmol/L) to simulate neuronal lesions characteristic of depressive illness. This approach would allow us to systematically evaluate whether FIASMA antidepressants uniquely confer cellular protection or if NO-FIASMA agents also exhibit such effects. *Does combining a FIASMA antidepressant with a NO-FIASMA antidepressant enhance antidepressant efficacy?* In a clinical scenario, adding a NO-FIASMA antidepressant (mianserine 60 mg/day) to the treatment of depressive patients who were unresponsive to a FIASMA antidepressant (fluoxetine 20 mg/day) resulted in a greater reduction in MADRS scores (-15 points) compared to fluoxetine monotherapy (-11 points) at six weeks (100). This observation is consistent with a meta-analysis of 39 combination studies (101), which generally report improved outcomes with dual antidepressant therapy. However, these studies typically compare combination treatments (dosage a+b) to monotherapies (dosage a only) and do not specifically address the unique interaction between FIASMA and NO-FIASMA properties. The question remains unresolved and warrants further investigation.

## Conclusion

Antidepressants simultaneously reduce the perception of danger in the host (mood effect) and promote a trophic state in cells. This trophic state is characterized by anti-inflammatory effects on the immune system, cytoprotective effects that enhance cell survival, increased host survival, reproductive effects on stem cells. These outcomes are partially mediated by the ASM/ceramide pathway and partially by the monoamine theory, which highlights their role in modulating membrane properties and synaptic cleft dynamics.
